# Synthesis of Iron Oxide Nanoparticle Functionalized Activated Carbon and Its Applications in Arsenic Adsorption

**DOI:** 10.1155/2021/6668490

**Published:** 2021-04-27

**Authors:** Hoang Thu Ha, Pham Tuan Phong, Tran Dinh Minh

**Affiliations:** ^1^VNU University of Education, Vietnam National University, Cau Giay, Hanoi 100000, Vietnam; ^2^High School for Gifted Students (HSGS), VNU University of Science, Hanoi 100000, Vietnam

## Abstract

This work reveals the As(V) adsorption behaviors onto iron oxide (Fe3O4) nanoparticles modified activated carbon (AC), originally developed from biochar (BC), as a green adsorbent denoted by FAC. Since FAC has abundant surface functional groups and a desired porous structure that is favorable for the removal of As(V) in contaminated water, FAC has greatly enhanced the As(V) adsorption capacity of the original BC. Various methods were employed to characterize the FAC characteristics and adsorption mechanism, including pH_pzc_ determination, BET specific surface area, elemental analysis (EA), and scanning electron microscopy (SEM). Results show that the AC surface was successfully modified by iron oxide nanoparticles, enhancing the porosity and specific surface area of original adsorbent. Batch adsorption tests indicated a well-fitted Langmuir model and pseudo-second-order model for As(V) adsorption. Additionally, the highest adsorption capacity (*Q*_max_ = 32.57 mg/g) by FAC was higher than previously reported literature reviews. Until now, no article was conducted to research the effect of carbon surface chemistry and texture on As removal from waters. It is required to obtain a rational view of optimal conditions to remove As from contaminated water.

## 1. Introduction

Recently, in many developing countries such as Laos, Vietnam, and Cambodia, Arsenic (As) has been detected as an emerging contaminant due to its detection in water environment and negative effects on human health, and natural ecosystem. As(V) is among abundant elements on Earth and participates in various natural processes as well as human activities. It has been identified as the king of poisons and the deadliest toxicity in the world, negatively affecting human health more than any other chemical elements in the periodic table [1, 2]. It was first discovered in the 8^th^ century by Jabir, an Arabian, in the form of a creative white arsenic or arsenic trioxide (As_2_O_3_). At that time, it was named product of Jabir, an odorless, tasteless toxic that could kill people very quickly without leaving any trace in the human body [3, 4]. The median lethal dose of LD_50_ (50% of laboratory tested animals have died) of arsenic is 763 mg per kilogram of body weight (via diet) and 13 mg per kilogram of body weight (via injection) [5]. However, arsenic is still not as toxic as its oxidizing compounds, such as arsenic trioxide (As_2_O_3_). As_2_O_3_ is around 500 times more deadly than elemental arsenic, similarly to dioxin. Arsenic is a naturally occurring component of the earth's crust, which is discharged into aquifers when water flows through the underlying sedimentary rock layers [6, 7]. Thus, the highest risk of As(V) pollution from drinking water in drilled wells and village drills is lower than that in lakes or streams. As and its compounds, once they penetrate into the livings, cannot be rejected through biochemical cycles and accumulated in body, causing serious impacts [8]. At present, it is estimated that more than 150 million people in worldwide have been affected by increasingly concentration of As in drinking water [9]. The most influenced areas that have been reported include Mekong delta (Vietnam, Cambodia), Hetao river (Mongolia), Duero Cenozoic Basin (Spain), and Tulare Lake (USA). Radical actions need to be done for treatment of As and its derivatives in water environment.

Iron oxide nanoparticles are metal oxide particles coming in the size range of approximately 10 to 100 nm. It is clearly proved that nanoscale Fe_3_O_4_ particles possess high magnetic nature, high surface area, electrical and thermal conductivity, and also excellent dimensional stability. The applications of iron oxide nanoparticles lie on multiple fields such as data storage and resonance imaging, alloy and catalyst industry, drug delivery, and medical treatment [10]. Currently, the iron oxide nanoparticles are among favorable choice for treatment of multiple ground contaminants, for example, polychlorinated biphenyls (PCBs), chlorinated organic solvents, and organochlorine pesticides. This feature is due to the fact that iron oxide nanoparticles are easily transportable through ground water, non-toxic to environment and human health, and thus can be injected into the polluted area and left to stay there for long periods of time [11]. The researchers demonstrated that aluminium and iron oxides are the most commonly applied materials for As removal [12, 13]. Dao et al. concluded that the nanocomposite of metal oxides (mixed oxide between iron and Mn_2_O_3_) with functionalized polymer of polyvinyl alcohol (PVA) are desired for the removal of As [14–16]. Mamindy-Pajany et al. investigated that the arsenic adsorption using activated iron minerals of iron such as goethite and haematite increased at acidic pH values [17]. Pena et al. observed that nanocrystal TiO_2_ powders displayed a great adsorption efficiency for As [18]. Currently, in Vietnam there are so many treatment facilities employed and applied to remove As in waters, especially in several cities such as Ha Nam, Thai Binh, Lam Dong, Ho Chi Minh, and Hanoi. According to the recent literature reviews, it shows that groundwater in these regions is contaminated with As at a high level, exceeding the allowed standard from 8 to 18 times. Therefore, the issue of clean water to ensure hygiene for people in Vietnam is not guaranteed [19].

In recent years, growing significance is contributed to the synthesis of biochar (BC) from biomass and wood waste (w.w), as an increase in research studies and publications can be found. It is a high-carbon source, fine-grained residue generated from the pyrolysis of agricultural and organic materials such as corncob, husk, stalk, potato, rice, and wheat straw, under the absence of oxygen. Interestingly, because of carbonization, BC had excellent physical (high surface area, porous morphology) and chemical composition (surface functional groups) associated with adsorptive capability. In comparison with currently commercial activated carbon (AC), BC has less defects, lower fabrication cost, and higher reusability. Furthermore, BC adsorption activity can be facilely enhanced by chemical activation or thermal modification. One of the current approaches is to modify carbon-based materials with active polymers by increasing the surface functional groups, resulting in improving As(V) adsorption efficiency such as [3-(2-aminoethylamino)propyl]trimethoxysilane (AEAPTMS) [15], (3-Aminopropyl)triethoxysilane (APTES) [20], and PVA [16]. The amino silane molecules of AEAPTMS are distributed on Fe_3_O_4_ magnetic nanoparticles via oxygen bridge. This is the reaction commonly used to attach organic functional groups to the surface of materials containing -OH groups on their surface [21]. Amine groups on the surface of Fe_3_O_4_ will serve as the basis for the fixation of surface complexes with As(V) in the next process. The advantage of magnetic materials is that both adsorbent and adsorbate are able to separate from the reaction system mixture easily by using a magnet. In addition, our materials are recoverable and reusable by depositing Fe_3_O_4_ on the surface of original biochar (OBC), thereby contributing to green chemistry-oriented treatment, and environmentally sustainable.

## 2. Methodology

### 2.1. Synthesis of Adsorbents OBC and FAC

#### 2.1.1. The Production of OBC

The technology to generate BC could be based on various pyrolysis platforms, which might be dated to thousands of years ago. In this study, the process of BC pyrolysis through the thermochemical route was derived from urban w.w at a high temperature range in the absence of oxygen. The collected w.w content is approximated in Table 1. As can be seen in Table 1, the w.w in type III obtained the highest mass fraction (wt%) of wood residues; therefore, it might be selected for further procedure. The carbon-rich source was used as the feedstock for the generation of OBC. Briefly, the w.w was collected in urban w.w in Hanoi, Vietnam, and then polysized in a quartz bowl inside a tubular furnace at a heating rate of 5°C/min, in ultra-high purity nitrogen (UHPN) atmosphere at a fixed temperature 500°C for 3 h. OBC was collected from the furnace and cooled to the room temperature (RT). After that, OBC was reduced to the extremely fine nanoparticle size, homogeneous mixture and mesoporous structure, and almost uniform distribution by using planetary ball-milling (or mechanical milling) process.

#### 2.1.2. Synthetic Route of Preparation of Fe_3_O_4_

Iron oxide nanoparticles were fabricated according to the previously published literatures with a small modification. In the first step, the following pure salts FeCl_3_.6H_2_O and FeCl_2_.4H_2_O with mole ratio of 1 : 2 were dissolved in ethanol (EtOH) at 95°C, and then the pH of suspension was stably adjusted up to 11 by gradually adding NaOH solution with the help of constant magnetic stirring for 40 min. The final solid was magnetically collected and then washed with deionized water (DI water) and EtOH in order to eliminate the unexpected impurity. Next, the solid was dried at 105°C in vacuum for further experiments.

#### 2.1.3. Synthesis of FAC

Fe_3_O_4_ nanoparticles and OBC were prepared as the starting materials via polymer-dispersible. In brief, about 1 g of Fe_3_O_4_, 5 g of OBC, and 300 mL EtOH were suspended in a round bottomed flask. It was sonicated at 65°C for 30 min, until the nanoparticles were dispersed completely. 15 mL AEAPTMS solution (technical grade, ≥97%) was drop-wise added to the flask under vigorous agitation. The mixture was then collected by a magnet and designated as FAC. About 1.5 g of FAC was carried out via thermal treatment was carried out in a quartz boat inside a horizontal tubular furnace, and heated to the favorable temperature of 600°C for 2 h. FAC was then cooled down over night in UHPN atmosphere. After the heat treatment, the final FAC was stored in a desiccator for further use and As(V) adsorption tests.

### 2.2. Material Characterizations

The BET specific surface area, total pore volume (TPV), and average pore diameter (APD) of OBC and FAC were examined by a Brunauer-Emmett-Teller (BET) analysis (NOVA, 2200E series), including total pore volume (TPV) and average porous diameter (APD). Surface science of the as-synthesized material was observed using field emission-scanning electron microscope (FE-SEM; JSM-F100). The main inorganic elemental analysis of materials was calculated by x-ray fluorescence (XRF analyzer, Perkin-Elmer). The determination of the point of zero charge (pH_pzc_) of each sample was conducted following the previous work [22]. Briefly, the pH of the solution in system was adjusted by adding either diluted HCl or NaOH solutions. A series of 50 mL of NaCl 0.01 M was prepared in pre-boiled water in order to prevent CO_2_ dissolution, and then, 0.1 mg of samples was added to 100 mL of I water with varying pH from 2 to 12 and constantly stirring for 24 h until the pH values reached stable. The final pH of the solution was plotted against initial pH of the solution. The initial and final concentrations of As(V) were measured by using inductively coupled plasma mass spectrometry (ICP-MS, model Agilent, 7500-Series, Japan).

### 2.3. Chemicals and Agents

All agents and As(V) standard solutions were of analytical grade and purchased from Sigma-Aldrich, and then stepwise diluted with DI water to obtain concentrations ranged within 5–100 mg/L.

### 2.4. Batch Adsorption Experiments

The batch adsorption experiments were performed within a series of 30 mg adsorbents mixed in range of 30–300 mL aqueous solution containing metallic As(V) ions at various concentrations ranged from 15 to 600 mg/L. The suspension containing the adsorbents and As(V) ions was shaken constantly until reaching to equilibrium state. All the tests were performed at 25°C and 120 rpm in a water bath.

### 2.5. As(V) Concentration Measurement

The percentage removal (R%) and the adsorption capacity *q*_*e*_ (mg/g) of adsorbents onto As(V) ions were calculated using equations (1) and (2) [23]:

(1) $$\begin{array}{c} R\mathrm{\%=}\frac{C_{o}-C_{e}}{C_{o}}\times 100\mathrm{\%,} \end{array}$$

(2) $$\begin{array}{c} q_{e}=\frac{\left(C_{o}-C_{e}\right)\times V}{m}, \end{array}$$

where *C*_*o*_ and *C*_*e*_, respectively, represent the initial and final concentrations of As(V) ions (mg/L), *V* is the volume of the solution (L), and *m* is the weight of adsorbent (mg).

## 3. Results

### 3.1. Sample Characterizations

#### 3.1.1. SEM Images and Textural Characteristics

**Figure 1:**
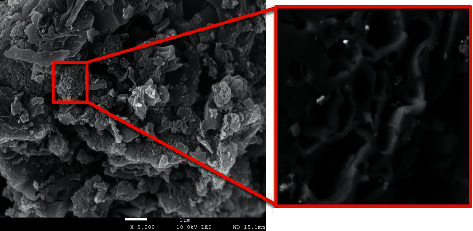
SEM images of FAC.

The particle morphology and surface characteristics of FAC were investigated by SEM analysis. As can be seen in Figure 1, the iron oxide incorporated activated carbon (FAC) displayed a fairly homogeneous surface with numerous tiny cracking, pores, and channels which helped improve the porosity of the FAC [24]. According to Table 2, the specific surface area BET, total pore volume (TPV), and average porous diameter (APD) of FAC are 998 m^2^·g^−1^, 1.12 cm^3^·g^−1^, and 2.7 nm, correspondingly. The formation of these pores and channels might be attributed to the carbonization of BC, the modification with iron oxide nanoparticles, the melting, and cracking processes, which altogether contributed to improve the porosity of the products [25, 26].

#### 3.1.2. TEM and XRD Studies

**Figure 2:**
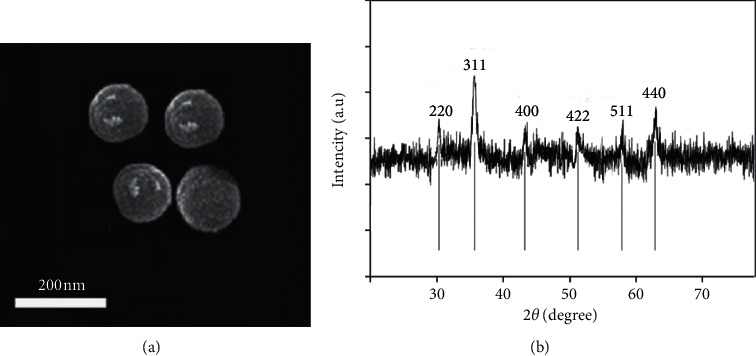
(a) TEM and (b) XRD results of FAC.

Figure 2(a) displays the TEM inner morphology of FAC composite with 200 nm scale bar. FAC has spherical shape, also describing the confirmation of uniform distribution of nano-sized Fe_3_O_4_ particles on AC surface. To analyze the crystal phase of FAC, the sample was determined by powder x-ray diffraction (XRD pattern) in the 2*θ* in area of 10–90° at 25°C by using Cu k*α* (*λ* = 1.54 Å) radiation. The characteristic studies using XRD peak in Figure 2(b) indicate the peak of FAC composite. It confirms that the highest peaks of Fe_3_O_4_ at 2*θ* value of 30.2, 35.5, 43.4, 53.5, 57.1, and 62.9° corresponded to (220), (311), (400), (422), (511), and (440) planes, compared with JCPDS No. 19-0629 [27].

#### 3.1.3. Inorganic Elemental Analysis

The elemental component of OBC and FAC samples (wt%) is shown in Table 3. The heat treatment highly increased the carbon content of FAC [28]. The carbon content increased from 42% (in OBC) to 65% (in FAC) along with the oxygen content decrease from 49.34% to 28.76% indicating the effect of carbonization in UHPN. The decreased content of H, N, and O of FAC might be due to dehydration process and decarboxylation related with heat treatment at high temperature [29].

#### 3.1.4. Determination of pH_pzc_ Values

**Figure 3:**
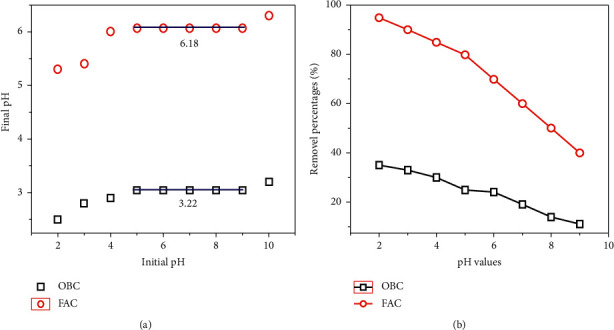
(a) Plot of final pH vs. initial pH, and (b) influences of pH on As(V) adsorption using OBC and FAC (*m* adsorbents = 50 mg; *V* As(V) solution = 50 mL; time = 30 min; *C*_*o*_ of As(V) = of 100 mg/L).

The pH_pzc_ is an important factor in the adsorption process for the adsorbents as it indicates the acidic or alkaline characteristic and the net surface charge of materials in aqueous solutions. pH_pzc_ is the pH at which the charge on the adsorbent surface is zero [30]. The pH_pzc_ value of OBC and FAC was calculated at 3.22 and 6.18, respectively (Figure 3(a)). These values indicate the acidic nature of both samples that was desired for As(V) removal because the positive network of material surfaces is attractive to the anionic As complexes [31]. The shift in pH_pzc_ of OBC and FAC from fairly strongly acidic 3.22 to nearly neutral 6.18 after modification implies the presence of alkaline functional groups (amino-NH_2_, -NH-groups of AEAPTMS) on the surface of the material. The role of AEAPTMS in this study is to create firmly chemical connection between iron oxide and OBC as well as to help distribute these nanoparticles evenly on the material surface.

### 3.2. Adsorption of As(V) Ions

#### 3.2.1. Influences of Solution pH

The pH factor of a solution is one of the most important factors affecting the uptake of metal ions. Generally, the acidic environment (pH range from 1 to 4) was the most beneficial condition for metallic anion uptake. Besides, pH above 8 might facilitate the precipitation of metal hydroxide. Therefore, in order to fully understand the effect of pH on the adsorption of As(V) onto OBC and FAC samples, experiments were conducted in a wide pH range of 2.0 to 9.0. As can be seen in Figure 3(b), both materials obtained the highest adsorption capacities (*q*_*e*_) of As(V) at the optimal pH ranged from 2 to 5 (acidic pH values), and *q*_*e*_ decreased in the alkaline medium (when pH > 6). It might be due to the fact that in the strong alkaline conditions (pH > 6) many OH^−^ ions can compete with As(V) anions for the adsorption sites of the adsorbent surface.

Arsenic is mainly presented in water environment in the form of aqueous anions and neutral molecules including HAsO_4_^2−^ (aq), H_2_AsO_4_^−^ (aq), and most likely partially as H_3_AsO_4_ (aq), AsO_4_^3−^ (aq), and H_2_AsO_3_^−^ (aq) in the pH ranged from 2 to 6. Meanwhile, as confirmation from Figure 2, the zeta potentials of OBC and FAC were 3.22 and 6.18, respectively. It means that while the solution pH < pH_pzc_, the surface charge of OBC and FAC is positive, and it is negative when pH > pH_pzc_. It is associated with the electrostatic interaction between surface sites and As(V) anion species in the solution, resulting in an enhanced adsorption capacity. For example, at the pH below 3, the surface charges of both OBC and FAC were positive, and the arsenic species existed in anion form, so it is reasonable to believe that the electrostatic interaction is the main mechanism in this process. If the pH of environmental solution is above 6, the OBC and FAC surface is charged negatively, so that the electrical repulsion strongly prevents the interaction of the adsorbent and the pollutant.

#### 3.2.2. Influence of Contact Time

**Figure 4:**
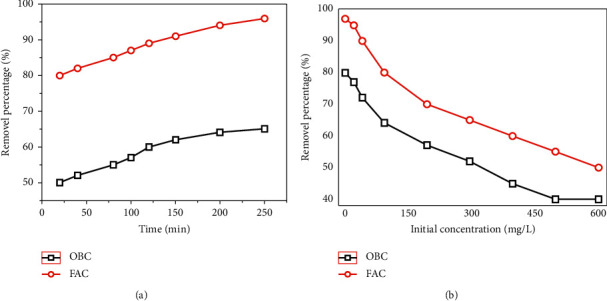
Effect of (a) contact time and (b) initial concentration on the As(V) adsorption by OBC and FAC.

The percentage removal of As(V) in the view of phase agitation time onto OBC and FAC is displayed in Figure 4(a). It might be investigated that the fast As(V) adsorption that occurred in the contact time ranged from 20 to 150 min, and then adsorption process became slow and almost maintained equilibrium after 180 min. It was because more active/available sites onto OBC and FAC were occupied by the adsorbed As(V) ions, and then the adsorption procedure was slowed down gradually. Lengthening the reaction time does not increase the uptake efficiency since the desorption rate reaches equal value to the adsorption rate. The obtained results in the present work can be explained that at the beginning of adsorption process As(V) is adsorbed by the specific sites of FAC, while with increasing adsorption time these specific sites are saturated to some extent and the exchange sites on the surface of FAC are fully filled by As molecular, leading to a maintained adsorption efficiency after 180 min.

#### 3.2.3. Effect of Initial Concentration of As(V) Standard Solutions

The initial concentration (*C*_*o*_) is another significant aspect affecting the As(V) adsorption (Figure 4(b)). It was revealed that when *C*_*o*_ increased, the As(V) removal percentage decreased dramatically, suggesting that As(V) adsorption is highly dependent on *C*_*o*_. As further discussed in the upcoming section, the monolayer adsorption of FAC favors low concentration of As ions. Since the detected concentrations of As(V) in actual environment are usually in level of *μ*gL^−1^, the uptake efficiency of As(V) onto FAC in actual application is predicted to be insignificantly different compared to laboratory conditions.

### 3.3. Adsorption Isotherms

**Figure 5:**
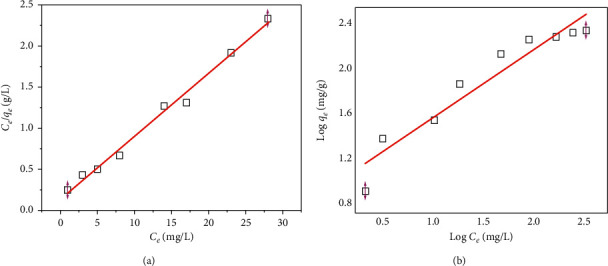
(a) Langmuir and (b) Freundlich plots on As(V) adsorption using FAC.

Adsorption isotherms play an essential role in the understanding of the adsorbed amount of As(V) removed from the aqueous solution by unit mass of adsorbents. They also provide important factors to design the adsorption system in the field. The Langmuir equation is expressed as

(3) $$\begin{array}{c} \frac{C_{e}}{q_{e}}=\frac{C_{e}}{q_{\text{max}}}+\frac{1}{K_{L}\cdot q_{\text{max}}}, \end{array}$$

where *q*_*e*_ is the amount of adsorbed As(V) ions per gram of adsorbent (mg/g), *q*_max_ is the maximum monolayer adsorption capacity (mg/g), *K*_*L*_ is the Langmuir constant related to the heat adsorption (L/mg), and *C*_*e*_ is the supernatant equilibrium concentration (mg/L). The Freundlich equation can be expressed as

(4) $$\begin{array}{c} \text{ln}q_{e}=\text{ ln}K_{F}+\;\frac{1}{n}\text{ln}C_{e}, \end{array}$$

where *K*_*F*_ is the Freundlich constant related with adsorption capacity (mg/g) and *n* the adsorption intensity, which is indicative of the bonding energy between As ions and adsorbent. These plots of Langmuir and Freundlich models are illustrated in Figure 5(a), and their parameters are calculated and listed in Table 4.

Based on the data given in Figure 5 and Table 4, the As(V) adsorption using FAC was well-fitted by the Langmuir model, better than that of Freundlich model. It suggested that the As(V) uptake behaviour on FAC is monolayer adsorption with homogeneous distribution of available networks on the surface of FAC. The value of 1/*n* in Freundlich model below 1 suggests a favorable level of As adsorption by FAC [32]. The *K*_*L*_ value of Langmuir model that ranged below 0.1 indicated a low surface energy in the adsorption process, thus revealing a stronger bonding force between As(V) ion and FAC [33, 34]. As can be seen in the table, the maximum adsorption capacity (*Q*_max_) of the FAC for As(V) ions, based on the Langmuir isotherm, was 32.57 mg/g, which was higher than that of other previously reported materials (Table 5), proving that FAC might be used as a highly promising adsorbent for As(V) removal in wastewater treatment.

### 3.4. Adsorption Kinetics

The adsorption kinetics of As(V) adsorption onto FAC, the pseudo-first-order and pseudo-second-order kinetic models were applied to the experimental data and then, respectively, expressed by equations (5) and (6):

(5) $$\begin{array}{c} \log\left(q_{e}-q_{t}\right)=\text{log}q_{e}-\frac{k_{\text{ad}}}{2.303}t, \end{array}$$

(6) $$\begin{array}{c} \frac{t}{q_{t}}=\;\frac{1}{h}+\;\frac{1}{q_{e}}\;t, \end{array}$$

where *q*_*e*_ and *q*_*t*_ (mg/g) represent the amount of the metal ions adsorbed at time *t* (min) and at equilibrium, respectively, *k*_ad_ is the rate constant of the adsorption (min^−1^), *h*=*kq*_*e*_^2^ (mg/g/min) is the initial sorption rate as *t*⟶0, and *k* represents the rate constant of pseudo-second-order adsorption (g/mg/min). These values were determined and are listed in Table 6. Two typical adsorption kinetic models, including pseudo-first-order and pseudo-second-order kinetic models, were applied to determine the kinetics of As(V) adsorption (Table 6). As can be seen in the table, the *q*_*e,* cal_ value of pseudo-first-order model was much lower than *q*_*e,* exp_, suggesting that the arsenic ion adsorption was satisfactorily matched to the pseudo-second-order model, while the pseudo-first-order kinetic model did not fit well the data. Table 6 also indicates that the well-fitting of As(V) adsorption resulting from the pseudo-second-order model yielded very high determination coefficients (*R*^2^ > 0.99) as compared with the pseudo-first order plot. It also indicated that the chemisorption as the rate-limiting mechanism took place during the adsorption by FAC surface rather than physisorption [2, 5, 19].

### 3.5. Proposal of Removal Mechanism

**Figure 6:**
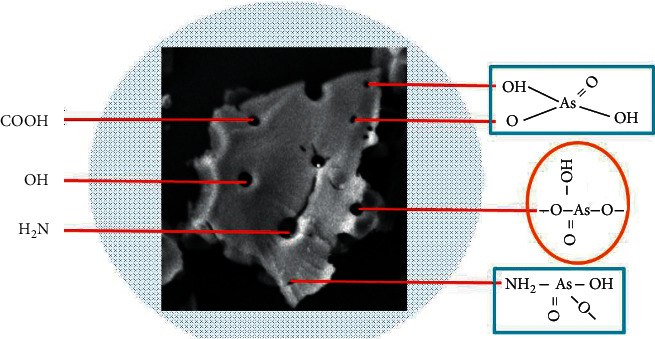
The proposed mechanism of As(V) adsorption by FAC.

According to experiment data on the adsorption kinetics and isotherms in this study, the authors proposed the potential removal mechanism for the As(V) as in Figure 6. In aqueous solution, the outermost iron atoms in iron oxide nanoparticles attract water molecules. After that, these water molecules are hydrolyzed to generate functional hydroxyl groups. Hence, the role of iron oxide nanoparticles is not only to increase the contacting surface area of the material but also to supplement numerous surface functional groups benefitting the removal. It is rational to believe that As^+5^ ions were mostly adsorbed on the surface of FAC via chemical interaction through the surface-bridging complexes by several functional groups onto FAC such as carboxyl (R-COOH^−^) groups, hydroxyl (R-OH^−^) groups, and amino (-NH_2_) groups. In outer sphere complex of FAC, these surface functional groups are coordinated and bound directly to the structural As^5+^, to form the surface complexation of FAC-As(V). Therefore, it was proposed to be a strong and easy adsorption of As(V) by FAC. There are few literature reviews of As(V) adsorption behaviors onto the adsorbent materials by ligand-exchange mechanism, where the As^+5^ cation will be exchanged with the surface functional groups of OH, COOH, OH, and NH_2_, which are directly incorporated to the FAC structural surface. That is the main reason for the higher removal rate of As(V) on the novel material FAC. Additionally, it is believed that the As(V) uptake onto FAC generates proton (H^+^ ions) to the solution, results in the pH value to strong acidic condition, and supports the adsorption equilibrium shift to the direction increasing removal efficiency. The mechanism totally agrees with observations in the pH investigation [21, 38].

## 4. Conclusions and Discussion

This work aimed to describe the facile synthesis of green bio-nanomaterial (FAC) from its precursor (BC from w.w) for As(V) adsorption. We figured out that the maximum adsorption capacity of FAC for As(V) was 32.57 mg/g in the views of adsorption kinetics and isotherms. The adsorption data demonstrated that the As(V) adsorption process can be matched by the pseudo-second-order kinetic equation and the adsorption isotherm was well-ﬁtted to the Langmuir model. The higher adsorption capacity of FAC for As(V) removal was also compared with the previously reported literatures. The effective studies of pH, time, and initial concentration confirmed that As(V) adsorption was favorably affected by the surface-bridging complexes, while As(V) was removed, associated with the ligand-exchange and electrostatic interaction.

## Figures and Tables

**Table 1:** Proximate analysis of urban w.w compositions and properties.

Content	w.w type I	w.w. type II	w.w. type III
Moisture (wt%)	11.8	12.4	13.5
Volatile (wt%)	85.4	77.6	83.9
Ash content (wt%)	2.3	3.1	3.7
Other waste matters (%)	1.2	1.1	2.0
Wood residues (%)	85.6	87.9	88.5

**Table 2:** The textural properties of FAC.

BET surface area (m^2^/g)	TPV (cm^3^/g)	APD (nm)
998	1.12	2.7

**Table 3:** The elemental analysis (wt%) of FAC and OBC products.

Samples	(%) C	(%) H	(%) N	(%) O
FAC	65.12	3.32	2.80	28.76
OBC	42.19	5.21	3.26	49.34

**Table 4:** The Langmuir and Freundlich model parameters.

Langmuir model			Freundlich model		
*q*_max_ (mg/g)	*K*_*L*_	*R* ^2^	*K*_F_	1/*n*	*R* ^2^
32.57	0.0766	0.98923	0.23	0.57	0.84

**Table 5:** The comparison of adsorption capacities of As(V) onto FAC and other adsorbents

Adsorbents	Adsorption capacities (mg/g)	pH	BET surface area (m^2^/g)	Diameter (nm)	References
Fe_3_O_4_ particles	16.6	5–7	179	10	[35]
Modified Fe/Mn-AC	19.35	3–6	973	2	[36]
Akaganeite	29.0	7	111	10–15	[37]
FAC	32.57	2–5	998	2.7	This study

**Table 6:** Kinetic adsorption parameters for As(V) adsorption.

*q*_*e*, exp_ (mg/g)	Pseudo-first-order model			Pseudo-second-order model			
	*k*_ad_ (min^−1^)	*q*_*e*, cal_ (mg/g)	*R* ^2^	*k* (g/mg/min)	*q*_*e*, cal_ (mg/g)	*R* ^2^	*h* (mg/g/min)
32.57	0.0072	12.47	0.841	0.031	34.86	0.999	8.356

Note: *q*_*e,* cal_: calculated maximum adsorption capacity; *q*_*e*, exp_: experimentally determined maximum adsorption capacity.

## Data Availability

All the data and supporting materials are included within the article.
