# EXPRESSION OF CONCERN: Synthesis of Iron Oxide Nanoparticle Functionalized Activated Carbon and Its Applications in Arsenic Adsorption

**DOI:** 10.1155/jamc/9762942

**Published:** 2026-05-21

**Authors:** Journal of Analytical Methods in Chemistry

EXPRESSION OF CONCERN: H. T. Ha, P. T. Phong, T. D. Minh, “Synthesis of Iron Oxide Nanoparticle Functionalized Activated Carbon and Its Applications in Arsenic Adsorption,” *Journal of Analytical Methods in Chemistry*, 2021, 6668490, https://doi.org/10.1155/2021/6668490.

This Expression of Concern is for the above article, published online on 28 April 2021 in Wiley Online Library (https://wileyonlinelibrary.com), and has been issued by agreement between the journal Editor‐in‐Chief, María José Trujillo‐Rodríguez; and John Wiley & Sons Ltd. The Expression of Concern has been agreed due to concerns raised on PubPeer [1] regarding the TEM image shown in Figure 2a, which shows the inner morphology of FAC composite nanoparticles. It appears that the representative nanoparticles shown are identical, with one of the three in the image field having been rotated 90 degrees.

The authors responded to a query by the publisher and supplied multiple SEM and TEM images of nanoparticles. They also explained that the particle characterization and imaging had been performed by an external laboratory. However, it could not be demonstrated unambiguously whether the particles shown in the raw data were of the same type as the ones shown in Figure 2a.

The Chief Editor and the Publisher are unable to make an unequivocal determination as to the morphology of the nanoparticles. Because the authors cannot provide any further data to better inform a decision, the journal has decided to issue an Expression of Concern in order to inform and alert readers.
